# Pharmacogenetic Approach for the Prevention of Rivaroxaban's ADRs: A Systematic Review and Meta-Analysis

**DOI:** 10.1155/2023/6105320

**Published:** 2023-10-31

**Authors:** Parham Mardi, Bahareh Abbasi, Arman Shafiee, Tara Afsharmoghaddam

**Affiliations:** ^1^Department of Medical Genetics, National Institute of Genetic Engineering and Biotechnology (NIGEB), Tehran, Iran; ^2^School of Medicine, Alborz University of Medical Sciences, Karaj, Iran; ^3^Faculty of Chemistry, Kharazmi University, Tehran, Iran

## Abstract

**Introduction:**

Pharmacogenetics is a potential approach that can be applied to decline the burden of rivaroxaban's ADRs. The current systematic review and meta-analysis aim to identify genetic variants correlated with rivaroxaban exposure and evaluate their importance.

**Methods:**

We systematically searched PubMed, Web of Science, and Scopus databases for all observational and interventional studies. The fixed effect method was used to pool the data when the Q-test's *p* value was higher than 0.1. We used random models when the *p* value was less than 0.1.

**Results:**

Data from ten studies (4721 participants) were analyzed in the current review. Qualitative synthesis from included studies found that two variants of ABCB1 (rs1045642 and rs2032582) and one variant of APOB (rs13306198) are potential contributors to rivaroxaban concentrations. Both wild homozygotes (AA) and heterozygotes (AC) of rs1045642 have significantly lower rivaroxaban concentrations compared to mutated homozygotes (CC) (SMD = 0.516, 95% CI: 0.115 to 0.917; SMD = 0.772, 95% CI: 0.088 to 1.455, respectively). Nevertheless, pooling unadjusted odds ratios did not yield a statistically significant correlation between rivaroxaban ADRs and genetic mutations.

**Conclusion:**

This study revealed that being an AC or CC for rs1045642 is attributed to a considerably higher rivaroxaban level in participants using rivaroxaban. That is to say, rs1045642 is a remarkable predictor of rivaroxaban metabolism. We concluded that identifying rs1045642 before drug administration might decrease ADRs although further studies adjusted for potential confounders are strongly suggested.

## 1. Introduction

Adverse drug reactions (ADRs) are hazardous reactions that result from using a medicinal product [1]. These unintended reactions burden patients [2–4]. Developed countries' healthcare systems undertake various strategies to reduce this burden, including educating clinicians and patients, enhancing platforms to report ADRs, providing ADR management guidelines, and producing safer drugs and antidotes [5–9]. Unlike strategies that focus on managing patients diagnosed with an ADR, methods such as pharmacogenetics concentrate on predicting an ADR before drug administration [10].

Pharmacogenetics is a well-established strategy that analyses how patients' genetic content influences drug metabolism [11, 12]. That is to say, amending the therapeutic approach based on the genetics of each patient leads to a lower risk of insufficient drug response and ADRs [13].

Both ADRs and insufficient drug responses of anticoagulants are potentially life-threatening [14]. Studies illustrated that although conventional anticoagulants such as warfarin have relatively lower efficacy than anti-Xa drugs such as rivaroxaban, rivaroxaban correlates with a higher risk of ADR, mainly gastrointestinal bleeding (GIB) [15]. As rivaroxaban is the oral anticoagulant of choice in low-income settings due to its reasonable price and cost-effectiveness, its prescription may impose a substantial burden on healthcare systems that are not prepared to manage an increased number of patients presenting with life-threatening bleeding. In other words, one of the requirements for the completion of the replacement of warfarin by rivaroxaban in the guidelines is to develop a reliable method to predict rivaroxaban's ADRs before drug administration [16]. The current study aims to perform a systematic review and meta-analysis to evaluate the efficacy of the pharmacogenetic approach in preventing rivaroxaban ADRs.

## 2. Methods

The current review was based on Preferred Reporting Items for Systematic Reviews and Meta-Analyses (PRISMA) [17].

### 2.1. Study Question

Is the pharmacogenetic approach effective in the prevention of rivaroxaban ADRs?

### 2.2. Search Strategy

Through this systematic search of original papers, under the approved protocol, title, abstract, and keywords of all observational and interventional studies, including cross-sectional studies, case-control, clinical trials, and cohorts, were searched in PubMed, Web of Science, and Scopus databases. To evaluate the efficacy of the pharmacogenetic approach in preventing rivaroxaban ADRs through systematic search, two independent researchers searched for relevant published and peer-reviewed scientific papers. The search terms were developed, concentrating on two primary roots of “rivaroxaban” and “genes, genetics, pharmacogenomics, pharmacogenetics, and personalized medicine.” There was no limitation on the paper's language and time of publication. For documents other than English and Persian, necessary arrangements were made for their specialized translation. The search strategy is demonstrated in Supplementary Table 1.

### 2.3. Inclusion Criteria

We included studies that considered rivaroxaban concentration, AUC, and ADR as the outcome in which cases with different genotypes were compared. Moreover, the included studies were case-control, cohort, clinical trial, and cross-sectional studies. We refined the searches for studies with human subjects without restrictions on language and publication year. Moreover, there was no limitation on the age of the participants in the studies. All nonrelevant publications or those that did not fit the abovementioned criteria were excluded. Furthermore, we also excluded all articles with duplicate citations.

### 2.4. Study Selection

Two independent researchers refined the relevant studies based on the inclusion criteria by going through three steps of data refinement, including titles, abstracts, and full-text review. A probable discrepancy between them was resolved by referencing the opinion of a third expert.

### 2.5. Data Management

The bibliographic information of the searched documents was saved on the EndNote software for further reference management. The required information was extracted and entered into Excel spreadsheets. Data collected according to a standard protocol, including data on citation information, type of study, sample size, exposure, outcome, age, and sex distribution of participants, were filled. Two independent researchers were involved in this process, and any probable discrepancy between them was resolved by referencing the opinion of a third expert.

### 2.6. Risk of Bias (Quality Assessment)

For observational studies, quality assessment was conducted using the Strengthening the Reporting of Observational Studies in Epidemiology (STROBE) statement consisting of a checklist comprising 22 items that researchers should consider when reporting observational studies [18]. Consolidated Standards of Reporting Trials (CONSORT), which consists of a checklist comprising 25 items, was used for the quality assessment of the included trial [19].

### 2.7. Data Analysis

The statistical analysis was carried out using Stata software, version 14. A *p* value of 0.05 or lower was considered statistically significant.

The fixed effect method was used to pool the data when the Q-test's *p* value was higher than 0.1. We used random models when the *p* value was less than 0.1.

The meta-analysis was performed when two or more studies reported similar exposures, outcomes, and confounding control. A forest plot was used to present the result of the meta-analysis schematically. Egger's test estimated publication bias.

## 3. Results

### 3.1. Systematic Review

**Figure 1:**
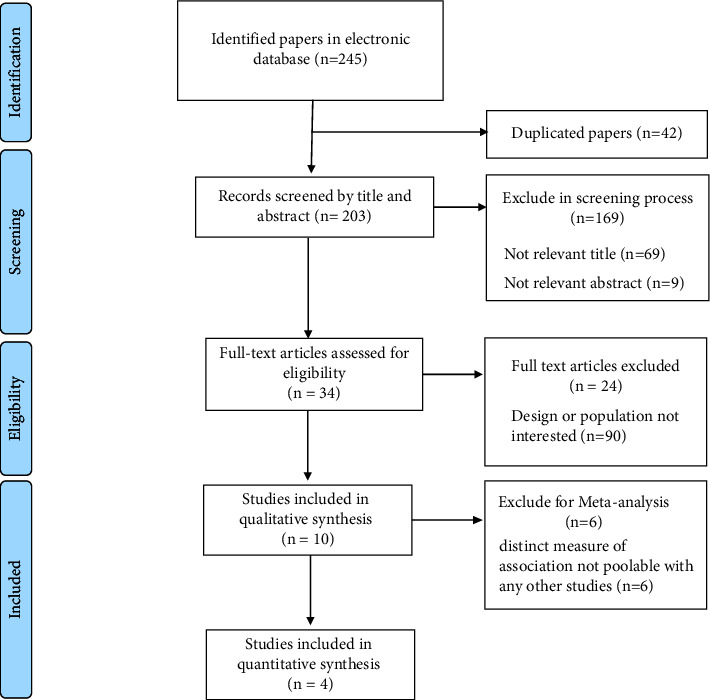
PRISMA flow diagram of included studies.

Overall, 245 records were yielded based on our search strategy. After removing duplicated studies and assessing studies based on their title, abstracts, and full texts, ten studies evaluating the correlation between genetic variants and rivaroxaban ADRs were included in our study.  Figure 1 demonstrates the PRISMA flow diagram of the systematic search.

### 3.2. Characteristics of Included Studies

Table 1 shows the characteristics of the included studies. Five of the included studies report data from patients presenting with atrial fibrillation. Also, four studies assessed patients receiving rivaroxaban due to atrial fibrillation or other medical indications. Gouin-Thibault et al.'s study is the only included study showing data from healthy volunteers. This record was the only study designed as a clinical trial. Eight and one of the included papers were cohorts and cross-sectional studies, respectively. Overall, data from 4721 participants (61.4% males) were included in this study.

### 3.3. Risk of Bias (Quality Assessment) Findings

Findings of quality assessment based on STROBE and CONSORT showed that all included studies were categorized into one group and had a high or relatively high quality (more than 16 out of 22 for descriptive studies and 21 out of 25 for the clinical trial). The results of the quality assessment are illustrated in Supplementary Table 2.

### 3.4. Qualitative Analysis

Our study illustrates the correlation of rivaroxaban ADRs or concentrations concerning genetic variants. Six genes (ABCB1, CYP3A4, CYP3A5, CYP2J2, ABCG2, and APOB) and fifteen mutations were evaluated in the current paper. To address the outcomes of the study, we extracted three main domains of variables: drug concentrations (maximum and minimum concentration), AUC (the area under the plasma drug concentration-time curve, which reflects body exposure to rivaroxaban), and ADRs (thrombotic and bleeding-related indices such as their incidence and prothrombin time).

As shown in Table 2, all unadjusted included studies reported nonsignificant data regarding the association of odds of ADRs and genetic variants. On the contrary, Yoon et al.'s study, which was the only adjusted study (adjusted for sex, age, overdose, rivaroxaban, anemia, and other genetic variants), demonstrated that being a carrier of rs1045642 or rs13306198 almost triples the odds of bleeding (OR for rs1045642 = 2.44, 95% CI: 1.07 to 5.58; OR for rs13306198 = 3.00, 95% CI: 1.39 to 6.47) [28]. Similarly, an adjusted prospective cohort indicated that the presence of rs1045642 decreases the hazard of a thromboembolic event by 58 percent (adjusted HR = 0.42, 95% CI: 0.18 to 0.98), which is a consequence of reduced rivaroxaban exposure [20].

Inline with the findings regarding ADRs, Sychev et al.'s study revealed that rs1045642 is correlated with a decreased maximum concentration of rivaroxaban [26] although data from Nakagawa et al.'s study did not indicate significant results [24].

Three studies considered AUC as the outcome of the association of rivaroxaban and genetic variants [21, 23, 25]. None of these studies demonstrated a significant link between genetic variants and rivaroxaban AUC.

### 3.5. Quantitative Analysis

Six records included in the qualitative synthesis reported distinct measures of association, which were not poolable with each other. Four studies were included in the quantitative synthesis. We undertook two approaches for meta-analysis. Initially, we included two records to evaluate genetic variants' effects on rivaroxaban concentration. In the second approach, in two other records, we assessed the effects of each variant on ADRs associated with rivaroxaban.

#### 3.5.1. Genetic Variants and Drug Concentrations

**Figure 2:**
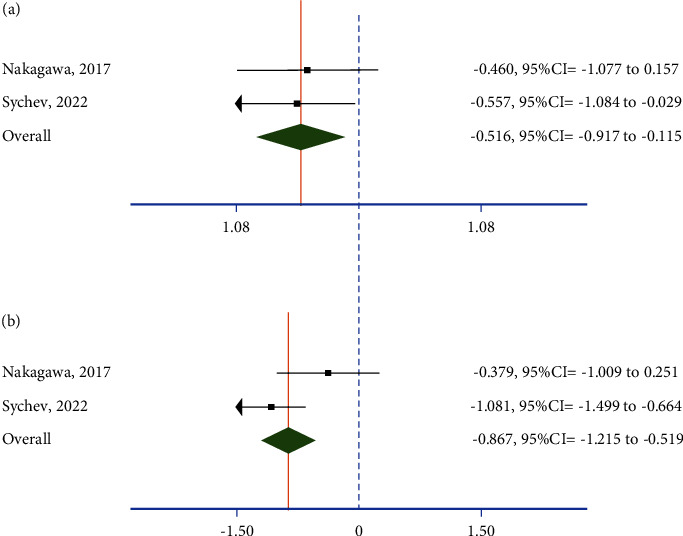
Forest plot of meta-analysis: (a) CC vs. AA and (b) AC vs. AA.

Our analysis based on pooling maximum concentration of rivaroxaban not only showed that CC of rs1045642 has significantly lower rivaroxaban concentration compared with AA (SMD = 0.516, 95% CI: 0.115 to 0.917) (Figure 2(a)) but also AC has significantly higher concentrations compared with AA (SMD = 0.772, 0.088 to 1.455) (Figure 2(b)). In other words, our findings showed that TT and CT patients have 12.92 ng/mL and 18.85 ng/mL lower concentrations than CC patients. Egger's test did not demonstrate a considerable publication bias (0.06, 95% CI: −0.02 to 0.14, *p* value =0.168). Meta-analysis results are summarized in Table 3.

#### 3.5.2. Genetic Variants and Odds of ADRs

As shown in Table 4, pooling unadjusted ORs showed that the odds of bleeding were not statistically different in carriers of rs2032582 or rs1045642. Egger's test did not show a considerable publication bias (−0.12, 95% CI: −0.32 to 0.08, *p* value =0.231).

## 4. Discussion

Our qualitative synthesis pointed out that two variants of ABCB1 (rs1045642 and rs2032582) and one variant of APOB (rs13306198) might contribute to drug concentration. As rs1045642 was eligible for meta-analysis, we followed our qualitative finding by pooling data from patients with similar rs1045642 genotypes. This quantitative synthesis submitted proof regarding the considerable association of rs1045642 (A3435C) and rivaroxaban concentration. We demonstrated that carriers of the C allele (CC and CA genotypes) on the 3435 position of ABCA1 have significantly higher rivaroxaban concentrations than participants with the AA genotype. However, our data are insufficient to claim that rs1045642 is attributed to a higher incidence of rivaroxaban ADRs.

That is to say, our raw findings indicated that while rs1045642 leads to an increment in rivaroxaban concentrations, it does not increase the risk of bleeding. We afresh reviewed included papers to identify the reason for this controversy.

We run into two possible explanations. First, we noticed that we extracted the ADR data from studies that were not adjusted for potential confounders, while studies on drug concentrations were adjusted for potential confounders. In other words, adjusting for confounders would alter our results, leading to a notable effect of rs1045642 on ADR. This hypothesis aligns with the findings of studies adjusted for potential confounders, which revealed that the incidence of bleeding is higher in carriers of rs1045642 [28, 29], while there is a lower hazard of thrombotic events [22].

Second, apart from administered dose, pharmacokinetic and genetic variants of drug concentrations, which are vital contributors to both drug concentration and its ADRs, other variables such as patient's age, the reason for drug administration, underlying disease, gender, and fasting condition also alter the risk of rivaroxaban's ADRs [16].

That is to say, we believe that rs1045642 in the ABCB1 gene might be a plausible candidate to be evaluated before rivaroxaban prescription, as ABCB1 not only predicts exposure and response to rivaroxaban [30, 31] but also is a notable contributor to the incidence and outcome of disease for which rivaroxaban is prescribed [32, 33].

Rivaroxaban is mainly prescribed to prevent stroke in nonvalvular atrial fibrillation (AF) and manage deep vein thrombosis (DVT) and pulmonary embolism (PE) [34].

Hypertension incidence, severity, and management are closely correlated with rs1045642 [35–37]. It also accounts for the most prevalent risk factor of AF and can worsen AF patients' prognosis [38]. These findings add to the importance of determining rs1045642 in AF patients treated with rivaroxaban. Like hypertension, malignancy is an independent criterion for diagnosing DVT [39], and rs1045642 contributes to chemotherapy response and overall survival in malignant patients [32, 40].

This overlap emphasizes the importance of rs1045642 screening, especially in low-income settings where other preventive and therapeutic strategies such as patient education, sequencing techniques, anti-Xa assays, and antidots are challenging, unavailable, or expensive [41, 42].

The assessment of the risk of bias in the included studies revealed that all included studies had a high or relatively high quality. In other words, the quality of included papers may not be a source of systematic error and it might not alter our meta-analysis results.

### 4.1. Strengths and Limitations

The limited number of available studies on this topic presents a significant constraint to our meta-analysis. Despite our extensive efforts in searching for relevant literature, the included papers represent the entirety of the available evidence. This limited number of studies may impact the generalizability of our findings and introduce potential bias. However, it is essential to acknowledge that including these studies has allowed us to comprehensively analyze the existing evidence and contribute to the current knowledge in this field. We believe that the current study can notably influence the field, so as to the best of our knowledge, it is the first study that systematically reviewed the impact of genetic variants on metabolism and risk of rivaroxaban ADRs. Our results provide a comprehensive overview of the current knowledge on rivaroxaban's pharmacogenetics that can be potentially beneficial in managing patients and stratifying their risk in the clinic. It should be considered that more original prospective high-quality studies are required to increase the certainty of our findings. However, as the first systematic review, the current paper proposes targets for future cohorts and trials.

## 5. Conclusion

The current study is the first meta-analysis that illustrated that being an AC or CC for rs1045642 is attributed to a considerably higher rivaroxaban level in participants using rivaroxaban. That is to say, rs1045642 is a remarkable predictor of rivaroxaban metabolism, and identification of rs1045642 before drug administration might decrease rivaroxaban ADRs. However, due to the limited number of available studies, data should be interpreted cautiously.

## Figures and Tables

**Table 1:** Characteristics of included studies.

Row	Author and year	Population	Provenance	Study design	Sample size	Age mean (SD)	Gender male (%)	Quality assessment
1	Campos-Staffico et al., 2022 [20]	Nonvalvular atrial fibrillation patients	United States	Retrospective cohort	2364	68.3 (13.6)	1606 (67.93)	19^*∗*^
2	Gouin-Thibault et al., 2016 [21]	Healthy male volunteers	France	Clinical trial	60	NR	60 (100.00)	21^*∗∗*^
3	Lähteenmäki et al., 2021 [22]	Finnish rivaroxaban users	Finland	Retrospective cohort	999	69.6 (9.8)	501 (50.15)	18^*∗*^
4	Lenoir et al., 2022 [23]	Hospitalized rivaroxaban users	Switzerland	Prospective cohort	125	71.7 (12.1)	89 (71.20)	18^*∗*^
5	Nakagawa et al., 2017 [24]	Nonvalvular atrial fibrillation	Japan	Prospective cohort	86	62.4 (10.6)	73 (84.88)	20^*∗*^
6	Sychev et al., 2019 [25]	Patients undergoing total hip and knee replacement surgery	Russia (Moscow)	Prospective cohort	78	59 (11)	22 (28.20)	19^*∗*^
7	Sychev et al., 2022 [26]	Patients aged 80 years and older with nonvalvular atrial fibrillation	Russia (Moscow)	Cross-sectional	128	87.5	NR	16^*∗*^
8	Wang et al., 2021 [27]	Patients with atrial fibrillation	Mongolia	Retrospective cohort	155	71.98 (10.72)	81 (52.25)	19^*∗*^
9	Yoon et al., 2022 [28]	Patients receiving direct oral anticoagulants	Korea	Retrospective cohort	576	NR	293 (50.86)	19^*∗*^
10	Zhang et al., 2023 [29]	Elderly patients with nonvalvular atrial fibrillation	Korea	Prospective cohort	150	68 (12.8)	63.33	17^*∗*^

NR, not reported; SD, standard deviation; ^*∗*^quality assessment based on STROBE statement; ^*∗∗*^quality assessment based on CONSORT statement.

**Table 2:** Qualitative analysis of included studies.

Row	Author, year	Exposure			Outcome definition	Measure of association and explanation	Results
		Gene name	Mutation	Grouping			
1	Campos-Staffico, 2022	CYP3A4	rs35599367	AA vs. AG vs. GG	Bleeding	HR (95% CI)	0.891 (0.708 to 1.122)
		CYP3A5	rs776746	CC vs. CT vs. TT			0.943 (0.687 to 1.294)
		CYP2J2	rs890293	CC vs. CA vs. AA			1.131 (0.871 to 1.468)
		ABCG2	rs2231142	GG vs. GA vs. AA			1.055 (0.863 to 1.289)
		ABCB1	rs4148732	GG vs. GA vs. AA			1.096 (0.956 to 1.256)
			C-G-C diplotypes	Homozygous vs. hetero vs. other			1.027 (0.895 to 1.179)

2	Gouin-Thibault, 2016	ABCB1	rs2677-3435	CC	AUC	Mean (percentage coefficient of variation)	1802, 42
			rs2677-3435	CT			2238, 43
			rs2677-3435	TT			2078, 50
			rs2677-3435	CC	Maximum concentration		161, 27
			rs2677-3435	CT			190, 31
			rs2677-3435	TT			178, 30

3	Lähteenmäki, 2021	ABCB1	rs1045642	CC vs. AA and AC	Bleeding	HR (95% CI)	0.84 (0.37 to 1.91)
			rs2032582	AA vs. AC and CC			1.06 (0.33 to 3.43)
			rs1128503	AA vs. AG and GG			1.03 (0.50 to 2.12)
			rs1045642	CC vs. AA and AC	Thromboembolic event		0.42 (0.18 to 0.98)^*∗*^
			rs2032582	AA vs. AC and CC			0.50 (0.23 to 1.08)
			rs1128503	AA vs. AG and GG			0.82 (0.36 to 1.89)

4	Lenoir, 2022	ABCB1	rs1128503	AG vs. AA	AUC	Mean (95% CI)	−46.50 (−163.59 to 70.59)
				GG vs. AA			21.46 (−125.94 to 168.86)
			rs1045642	AC vs. CC			−51.69 (−170.92 to 67.54)
				AA vs. CC			−71.90 (−161.27 to 17.46)
			rs2032582	GT vs. GG			56.52 (−75.24 to 188.29)
				TT vs. GG			54.86 (−96.09 to 205.81)

5	Nakagawa, 2017	CYP3A5	rs776746	TT	Maximum concentration	Mean (95% CI)	4.77 (3.83 to 7.36)
				TC			3.58 (2.21 to 5.13)
				CC			2.99 (1.94 to 5.34)
		ABCB1	rs1045642	AA			2.87 (2.45 to 5.14)
				AC			3.43 (1.89 to 5.72)
				CC			3.76 (2.12 to 5.14)
			rs2032582	GG			4.17 (2.16 to 6.05)
				GT			3.17 (1.31 to 5.11)
				TT			3.35 (2.45 to 5.41)
			rs1128503	AA			4.37 (2.49 to 4.91)
				AG			3.09 (2.11 to 5.72)
				GG			3.06 (2.18 to 5.30)
		ABCG2	rs2231142	GG			3.35 (2.25 to 5.14)
				GA			3.47 (1.88 to 5.39)
				AA			1.89 (0.99 to 3.49)
		CYP2J2	rs890293	CC			3.26 (2.11 to 5.14)
				CA			4.31 (2.39 to 8.60)
				AA			NR

6	Sychev, 2019	ABCB1	rs1045642	AA	AUC	Mean, *p* to value	150.4
				AC			121, 0.306
				CC			128, 0.857
			rs4148738	CC			142.2
				CA			120.8, 0.391
				AA			131.3, 0.987
		CYP3A4	rs35599367	GG			123.5
				GA			118.2, 0.716
				AA			NR
		CYP3A5	rs776746	TT			193.3
				TC			121.0, 0.217
				CC			NR

7	Sychev, 2022	ABCB1	rs1045642	AA	Maximum concentration	Mean (95% CI)	57.7 (23.3 to 75.8)
				AC			50.6 (28.3 to 80.6)
				CC			65.8 (36.4 to 95.7)
				AA	Gastrointestinal bleeding	Number of patients presented with outcome/genotype (%)	0/22 (0)
				AC			3/65 (4.6)
				CC			1/41 (2.4)
				AA	Hematuria		0/22 (0)
				AC			6/65 (9.2)
				CC			9/41 (22)
				AA	Prothrombin time	Mean (95% CI)	13.3 (12.4 to 14.5)
				AC			14.0 (12.6 to 14.8)
				CC			14.2 (13.0 to 16.1)
			rs4148738	CC	Maximum concentration	Mean (95% CI)	57.7 (28.3 to 98.0)
				CA			52.1 (28.4 to 80.4)
				AA			56.35 (36.3 to 89.1)
				CC	Gastrointestinal bleeding	Number of patients presented with outcome/each genotype (%)	1/37 (2.7)
				CA			2/63 (3.2)
				AA			1/28 (3.6)
				CC	Hematuria		1/37 (2.7)
				CA			5/63 (7.9)
				AA			9/28 (32.1)
				CC	Prothrombin time	Mean (95% CI)	13.4 (12.4 to 14.7)
				CA			14.0 (12.8 to 14.6)
				AA			14.2 (12.9 to 16.5)

8	Wang, 2021	ABCB1	rs1045642	AA	Bleeding	Number of patients in each genotype, presented with bleeding	22, 3
				AC			70, 10
				CC			63, 11
			rs1128503	AA			65, 0
				AG			63, 10
				GG			27, 5
			rs4148738	CC			31, 3
				CA			64, 10
				AA			60, 11

9	Yoon, 2022	ABCB1	rs1045642	AA vs. AC and CC	Bleeding	OR (%)	2.44 (1.07 to 5.58)^*∗*^
		APOB	rs693	TT vs. CC and CT			6.85 (0.90 to 52.63)
			rs13306198	GG vs. AA and GA			3.00 (1.39 to 6.47)^*∗*^

10	Zhang, 2023	ABCB1	rs4728709	GG vs. AA and GA	Increase in maximum concentration	Mean (SD)	47.6% (9.1%)

HR, hazard ratio; OR, odds ratio; CI, confidence interval; AUC, area under the plasma concentration-time curve.

**Table 3:** Impact of rs1045642 on rivaroxaban concentrations.

Description of analysis	Pooled SMD (95% CI)	Sample size	Heterogeneity		
			*I* ^2^ (%)	p value	Model
CC vs. AA	−0.516 (−0.917 to −0.115)	86	0.0	0.815	Random
AC vs. AA	−0.867 (−1.215 to −0.519)	86	69.8	0.069	Fixed

SMD, standardized mean difference; CI, confidence interval; *p* value was reported for the heterogeneity chi-squared test. A fixed model was used whenever *p* value was less than 0.1.

**Table 4:** Genetic mutations in odds of rivaroxaban ADRs (bleeding following rivaroxaban administration).

Gene	Mutation	Description of analysis	Pooled OR (95% CI)	Heterogeneity		
				*I* ^2^ (%)	p value	Model
ABCB1	rs1045642	CC vs. AA	5.46 (−5.15 to 16.08)	67.5	0.079	Fixed
ABCB1	rs1045642	AC and CC vs. AA	2.71 (−1.90 to 7.34)	0.0	0.384	Random
ABCB1	rs4148738	AA vs. CC	2.95 (−1.69 to 7.59)	0.0	0.385	Random
ABCB1	rs4148738	AC and AA vs. CC	3.08 (−6.89 to 13.06)	70.5	0.066	Fixed

ADR, adverse drug reaction; OR, odds ratio; CI, confidence interval; *p* value was reported for the heterogeneity chi-squared test. A fixed model was used whenever *p* value was less than 0.1.

## Data Availability

The data used to support the findings of this study are included within the article.

## Supplementary Materials

Supplementary Materials Supplementary Table 1: search strategy. This table provides a detailed description of the search strategy used to retrieve relevant articles for the systematic review and meta-analysis of the pharmacogenetic approach for preventing adverse drug reactions (ADRs) associated with rivaroxaban. Supplementary Table 2: quality assessment of included studies. This supplementary table presents the quality assessment of the studies included in the systematic review and meta-analysis. Quality assessment was conducted based on two different criteria, the STROBE (Strengthening the Reporting of Observational Studies in Epidemiology) statement for descriptive studies and the CONSORT (Consolidated Standards of Reporting Trials) statement for clinical trials.
