# Supplementary material for: Pharmacogenetic Approach for the Prevention of Rivaroxaban's ADRs: A Systematic Review and Meta-Analysis
Source: Genet Res (Camb). 2023 Oct 31;2023:6105320. doi: 10.1155/2023/6105320 (PMC10630013; doi:10.1155/2023/6105320)
Supplement: Supplementary Materials — Supplementary Table 1: search strategy. This table provides a detailed description of the search strategy used to retrieve relevant articles for the systematic review and meta-analysis of the pharmacogenetic approach for preventing adverse drug reactions (ADRs) associated with rivaroxaban. Supplementary Table 2: quality assessment of included studies. This supplementary table presents the quality assessment of the studies included in the systematic review and meta-analysis. Quality assessment was conducted based on two different criteria, the STROBE (Strengthening the Reporting of Observational Studies in Epidemiology) statement for descriptive studies and the CONSORT (Consolidated Standards of Reporting Trials) statement for clinical trials. [file 6105320.f1.zip › sup table 1 (1).docx]

| **Supplementary table 1. Search Strategy** | |
| --- | --- |
| PubMed | (((rivaroxaban[MeSH Terms])) AND (“Pharmacogenetics” )) OR ( ((rivaroxaban[MeSH Terms])) AND (“Pharmacogenomics” )) OR ( ((rivaroxaban[MeSH Terms])) AND (“drug response” )) OR ( ((rivaroxaban[MeSH Terms])) AND (“gene” )) OR ( ((rivaroxaban[MeSH Terms])) AND (“CYP” )) OR ( ((rivaroxaban[MeSH Terms])) AND (“ABCB1”)) |
| Web of Science (WoS) | ((“5-chloro-N-(((5S)-2-oxo-3-(4-(3-oxomorpholin-4-yl)phenyl)-1,3-oxazolidin-5-yl)methyl)thiophene-2-carboxamide” ) AND (“Pharmacogenetics” )) OR ( (“5-chloro-N-(((5S)-2-oxo-3-(4-(3-oxomorpholin-4-yl)phenyl)-1,3-oxazolidin-5-yl)methyl)thiophene-2-carboxamide” ) AND (“Pharmacogenomics” )) OR ( (“5-chloro-N-(((5S)-2-oxo-3-(4-(3-oxomorpholin-4-yl)phenyl)-1,3-oxazolidin-5-yl)methyl)thiophene-2-carboxamide” ) AND (“drug response” )) OR ( (“5-chloro-N-(((5S)-2-oxo-3-(4-(3-oxomorpholin-4-yl)phenyl)-1,3-oxazolidin-5-yl)methyl)thiophene-2-carboxamide” ) AND (“gene” )) OR ( (“5-chloro-N-(((5S)-2-oxo-3-(4-(3-oxomorpholin-4-yl)phenyl)-1,3-oxazolidin-5-yl)methyl)thiophene-2-carboxamide” ) AND (“CYP” )) OR ( (“5-chloro-N-(((5S)-2-oxo-3-(4-(3-oxomorpholin-4-yl)phenyl)-1,3-oxazolidin-5-yl)methyl)thiophene-2-carboxamide” ) AND (“ABCB1”)) OR ( (“ Xarelto” ) AND (“Pharmacogenetics” )) OR ( (“ Xarelto” ) AND (“Pharmacogenomics” )) OR ( (“ Xarelto” ) AND (“drug response” )) OR ( (“ Xarelto” ) AND (“gene” )) OR ( (“ Xarelto” ) AND (“CYP” )) OR ( (“ Xarelto” ) AND (“ABCB1”)) OR ( (“BAY 59-7939” ) AND (“Pharmacogenetics” )) OR ( (“BAY 59-7939” ) AND (“Pharmacogenomics” )) OR ( (“BAY 59-7939” ) AND (“drug response” )) OR ( (“BAY 59-7939” ) AND (“gene” )) OR ( (“BAY 59-7939” ) AND (“CYP” )) OR ( (“BAY 59-7939” ) AND (“ABCB1”)) OR ( (“rivaroxaban”) AND (“Pharmacogenetics” )) OR ( (“rivaroxaban”) AND (“Pharmacogenomics” )) OR ( (“rivaroxaban”) AND (“drug response” )) OR ( (“rivaroxaban”) AND (“gene” )) OR ( (“rivaroxaban”) AND (“CYP” )) OR ( (“rivaroxaban”) AND (“ABCB1”)) |
| Scopus | ((“5-chloro-N-(((5S)-2-oxo-3-(4-(3-oxomorpholin-4-yl)phenyl)-1,3-oxazolidin-5-yl)methyl)thiophene-2-carboxamide” ) AND (“Pharmacogenetics” )) OR ( (“5-chloro-N-(((5S)-2-oxo-3-(4-(3-oxomorpholin-4-yl)phenyl)-1,3-oxazolidin-5-yl)methyl)thiophene-2-carboxamide” ) AND (“Pharmacogenomics” )) OR ( (“5-chloro-N-(((5S)-2-oxo-3-(4-(3-oxomorpholin-4-yl)phenyl)-1,3-oxazolidin-5-yl)methyl)thiophene-2-carboxamide” ) AND (“drug response” )) OR ( (“5-chloro-N-(((5S)-2-oxo-3-(4-(3-oxomorpholin-4-yl)phenyl)-1,3-oxazolidin-5-yl)methyl)thiophene-2-carboxamide” ) AND (“gene” )) OR ( (“5-chloro-N-(((5S)-2-oxo-3-(4-(3-oxomorpholin-4-yl)phenyl)-1,3-oxazolidin-5-yl)methyl)thiophene-2-carboxamide” ) AND (“CYP” )) OR ( (“5-chloro-N-(((5S)-2-oxo-3-(4-(3-oxomorpholin-4-yl)phenyl)-1,3-oxazolidin-5-yl)methyl)thiophene-2-carboxamide” ) AND (“ABCB1”)) OR ( (“ Xarelto” ) AND (“Pharmacogenetics” )) OR ( (“ Xarelto” ) AND (“Pharmacogenomics” )) OR ( (“ Xarelto” ) AND (“drug response” )) OR ( (“ Xarelto” ) AND (“gene” )) OR ( (“ Xarelto” ) AND (“CYP” )) OR ( (“ Xarelto” ) AND (“ABCB1”)) OR ( (“BAY 59-7939” ) AND (“Pharmacogenetics” )) OR ( (“BAY 59-7939” ) AND (“Pharmacogenomics” )) OR ( (“BAY 59-7939” ) AND (“drug response” )) OR ( (“BAY 59-7939” ) AND (“gene” )) OR ( (“BAY 59-7939” ) AND (“CYP” )) OR ( (“BAY 59-7939” ) AND (“ABCB1”)) OR ( (“rivaroxaban”) AND (“Pharmacogenetics” )) OR ( (“rivaroxaban”) AND (“Pharmacogenomics” )) OR ( (“rivaroxaban”) AND (“drug response” )) OR ( (“rivaroxaban”) AND (“gene” )) OR ( (“rivaroxaban”) AND (“CYP” )) OR ( (“rivaroxaban”) AND (“ABCB1”)) |
| Filters | No language restriction; from inception, until March 1, 2023; |
