# Supplementary material for: Pharmacogenetic Approach for the Prevention of Rivaroxaban's ADRs: A Systematic Review and Meta-Analysis
Source: Genet Res (Camb). 2023 Oct 31;2023:6105320. doi: 10.1155/2023/6105320 (PMC10630013; doi:10.1155/2023/6105320)
Supplement: Supplementary Materials — Supplementary Table 1: search strategy. This table provides a detailed description of the search strategy used to retrieve relevant articles for the systematic review and meta-analysis of the pharmacogenetic approach for preventing adverse drug reactions (ADRs) associated with rivaroxaban. Supplementary Table 2: quality assessment of included studies. This supplementary table presents the quality assessment of the studies included in the systematic review and meta-analysis. Quality assessment was conducted based on two different criteria, the STROBE (Strengthening the Reporting of Observational Studies in Epidemiology) statement for descriptive studies and the CONSORT (Consolidated Standards of Reporting Trials) statement for clinical trials. [file 6105320.f1.zip › supp table 2 (1).docx]

| **Supplementary Table 2.**  **Quality Assessment of included studies.** | | | | | | | | | | | | | | | | | | | | | | | | | |
| --- | --- | --- | --- | --- | --- | --- | --- | --- | --- | --- | --- | --- | --- | --- | --- | --- | --- | --- | --- | --- | --- | --- | --- | --- | --- |
| **Descriptive studies**  **(Quality Assessment based on STROBE statement)** | | | | | | | | | | | | | | | | | | | | | | | | | |
| row | Author, year | Study design | 1 | 2 | 3 | 4 | 5 | 6 | 7 | 8 | 9 | 10 | 11 | 12 | 13 | 14 | 15 | 16 | 17 | 18 | 19 | 20 | 21 | 22 | Quality Assessment |
| 1 | Campos-Staffico, 2022  (20) | Retrospective cohort |  |  |  |  |  |  |  |  |  |  |  |  |  |  |  |  |  |  |  |  |  |  | 19* |
| 2 | Lähteenmäki,2021  (22) | Retrospective cohort |  |  |  |  |  |  |  |  |  |  |  |  |  |  |  |  |  |  |  |  |  |  | 18* |
| 3 | Lenoir,2022  (23) | Prospective cohort |  |  |  |  |  |  |  |  |  |  |  |  |  |  |  |  |  |  |  |  |  |  | 18* |
| 4 | Nakagawa,2017  (24) | Prospective cohort |  |  |  |  |  |  |  |  |  |  |  |  |  |  |  |  |  |  |  |  |  |  | 20* |
| 5 | Sychev,2019  (25) | Prospective cohort |  |  |  |  |  |  |  |  |  |  |  |  |  |  |  |  |  |  |  |  |  |  | 19* |
| 6 | Sychev,2022  (26) | Cross-sectional |  |  |  |  |  |  |  |  |  |  |  |  |  |  |  |  |  |  |  |  |  |  | 16* |
| 7 | Wang,2021  (27) | Retrospective cohort |  |  |  |  |  |  |  |  |  |  |  |  |  |  |  |  |  |  |  |  |  |  | 19* |
| 8 | Yoon,2022  (28) | Retrospective cohort |  |  |  |  |  |  |  |  |  |  |  |  |  |  |  |  |  |  |  |  |  |  | 19* |
| 9 | Zhang,2023  (29) | Prospective cohort |  |  |  |  |  |  |  |  |  |  |  |  |  |  |  |  |  |  |  |  |  |  | 17* |

| **Descriptive study**  **(Quality Assessment based on CONSORT statement)** | | | | | | | | | | | | | | | | | | | | | | | | | | | | |
| --- | --- | --- | --- | --- | --- | --- | --- | --- | --- | --- | --- | --- | --- | --- | --- | --- | --- | --- | --- | --- | --- | --- | --- | --- | --- | --- | --- | --- |
| row | Author, year | Study design | 1 | 2 | 3 | 4 | 5 | 6 | 7 | 8 | 9 | 10 | 11 | 12 | 13 | 14 | 15 | 16 | 17 | 18 | 19 | 20 | 21 | 22 | 23 | 24 | 25 | Quality Assessment |
| 1 | Gouin-Thibault, 2016  (21) | Clinical trial |  |  |  |  |  |  |  |  |  |  |  |  |  |  |  |  |  |  |  |  |  |  |  |  |  | 21** |
| Green: Study fulfills the criterion.  Red: Study does not fulfill the criterion. | | | | | | | | | | | | | | | | | | | | | | | | | | | | |
